# Radiological characterization of the tailings of an abandoned copper mine using a neural network and geostatistical analysis through the Co-Kriging method

**DOI:** 10.1007/s10653-024-02070-8

**Published:** 2024-07-09

**Authors:** V. M. Expósito-Suárez, J. A. Suárez-Navarro, A. Caro, M. B. Sanz, G. Hernaiz, A. González-Sanabria, M. J. Suárez-Navarro, L. Jordá-Bordehore, H. Chamorro-Villanueva, M. Arlandi, J. F. Benavente

**Affiliations:** 1 Centro de Investigaciones Energéticas, Medioambientales y Tecnológicas (CIEMAT), Avenida Complutense 40, 28040 Madrid, Spain; 2 Departamento de Hidráulica, Energía y Medioambiente, E.T.S.I. Caminos, Universidad Politécnica de Madrid (UPM), Canales y Puertos, Profesor Aranguren S/N, 28040 Madrid, Spain; 3 Sociedad Española Para La Defensa del Patrimonio Geológico y Minero (SEDPGYM), C/ Ríos Rosas, 21 28003 Madrid, Spain; 4 Túneles y Geomecánica S.L. C/ Calle Alfonso Gómez nº 17, 28037 Madrid, Spain

**Keywords:** Ambient dose equivalent, Co-Kriging, Geostatistics, Mine dump, Natural radioactivity, Neural network, Uranium series

## Abstract

The radiological characterization of soil contaminated with natural radionuclides enables the classification of the area under investigation, the optimization of laboratory measurements, and informed decision-making on potential site remediation. Neural networks (NN) are emerging as a new candidate for performing these tasks as an alternative to conventional geostatistical tools such as Co-Kriging. This study demonstrates the implementation of a NN for estimating radiological values such as ambient dose equivalent (H*(10)), surface activity and activity concentrations of natural radionuclides present in a waste dump of a Cu mine with a high level of natural radionuclides. The results obtained using a NN were compared with those estimated by Co-Kriging. Both models reproduced field measurements equivalently as a function of spatial coordinates. Similarly, the deviations from the reference concentration values obtained in the output layer of the NN were smaller than the deviations obtained from the multiple regression analysis (MRA), as indicated by the results of the root mean square error. Finally, the method validation showed that the estimation of radiological parameters based on their spatial coordinates faithfully reproduced the affected area. The estimation of the activity concentrations was less accurate for both the NN and MRA; however, both methods gave statistically comparable results for activity concentrations obtained by gamma spectrometry (Student's t-test and Fisher's F-test).

**Supplementary Information:**

The online version contains supplementary material available at 10.1007/s10653-024-02070-8.

## Introduction

Abandoned mine tailings are an environmental concern due to the potential migration of large quantities of heavy metals into surrounding aquifers. In addition, these tailings may include natural radionuclides due to the geological formation of the terrain. The initial problem arising from the presence of heavy metals is, therefore, also a radiological problem. An increase in the activity concentration of natural radioactive series radionuclides above background levels increases environmental doses; this must be assessed in order to prevent their dispersion and, if necessary, to remediate the affected area (Momčilović et al., 2013). The characterisation of mining-affected areas is typically done by plotting *in-situ* values using georeferencing techniques and estimated values through kriging y cokriging models (Duarte et al., 2022; Ruiz-Roso et al., 2020; Stanković et al., 2016). The maps developed in previous studies of tailings from an abandoned uranium mine have allowed us to identify areas of higher radionuclide presence and even to elucidate patterns of radionuclide migration due to wind (Gil-Pacheco et al., 2020). Similarly, previous studies have estimated ^137^Cs deposition in soils of the Iberian Peninsula (Caro et al., 2013) using geostatistical models. Additionally, it is possible to predict the activity concentration of radionuclides present in tailings by correlating *in-situ* measurements with laboratory determinations using linear regression (Carvalho et al., 2007).

Neural networks (NNs) have been successfully applied to mining exploration, extraction and logistics management processes (Baek & Choi, 2020; Caro et al., 2013). Similarly, they can be used to utilise the minerals contained in mine tailings (Cai et al., 2022). Other applications include terrain characterisation for remediation purposes (Ewing et al., 2023) and studies related to contaminant migration from mine tailings (Shukla et al., 2023). These applications involve minerals containing stable heavy metals, rather than those that may have a higher radioactive content. In addition, other studies have used NN to determine natural radionuclides in geological materials (Erzin & Yaprak, 2022). Therefore, in this work, we aim to verify the effectiveness of a NN for radiologically characterising a mine tailing site, as well as its use for estimating the activity concentration of radionuclides belonging to the uranium, actinium, and thorium radioactive series.

Based on the above considerations, our working hypothesis was that neural networks (NNs) allow the radiological characterisation of a tailings pile with high concentrations of natural radionuclide activity, providing results equivalent to those obtained by geostatistical software such as ArcGIS (Co-Kriging) in combination with multiple regression analysis. We tested our hypothesis by means of the following sub-objectives: i) correlation of field radiological measurements (ambient dose equivalent H*(10) and surface activity) with laboratory measurements (gross alpha and beta activity indices and gamma spectrometry); ii) development of two NNs, the first to estimate radiological field values from spatial coordinates and, based on these, a second NN to estimate activity concentrations of radionuclides from the uranium, actinium and thorium series; and iii) a comparison of the accuracy and precision of the results obtained using NNs with those obtained by ArcGIS (Co-Kriging) together with multivariate regression analysis.

## Experimental

### Study area

The Antigua Pilar mine is located in the municipality of Colmenarejo in the Community of Madrid, Spain. The mine was exploited for copper ore and closed in 1912. However, the mine was also known as a uranium mine due to the presence of the minerals torbernite (Cu(UO_2_)_2_(PO_4_)_2_·8-12H_2_O) and zeunerite (Cu(UO_2_)_2_(AsO_4_)_2_·12H_2_O), which are associated with high-concentration uranium activity (Chamorro et al., 2023). Other minerals present in the dump included chalcopyrite (CuFeS_2_), arsenopyrite (FeAsS), malachite (Cu_2_CO_3_(OH)_2_), azurite (Cu_3_CO_3_(OH)_2_), siderite (FeCO_3_), fluorite (CaF_2_), olivenite (Cu_2_AsO_4_(OH)) and chalcanthite (Cu_2_(SO_4_)·5H_2_O) (Pérez-Esteban et al., 2019). The geology of the mine was exposed to hydrothermal alteration, resulting in the natural radioactive series of uranium being several orders of magnitude higher than that of thorium (Galindo et al., 1994). In addition, heavy machinery was used to remove the tailings in 2020, resulting in significant changes to the terrain. Due to all of the above factors, the presence of uranium series radionuclides in the tailings and the alteration of the terrain provide a unique opportunity to undertake the characterisation proposed in this work.

**Fig. 1:**
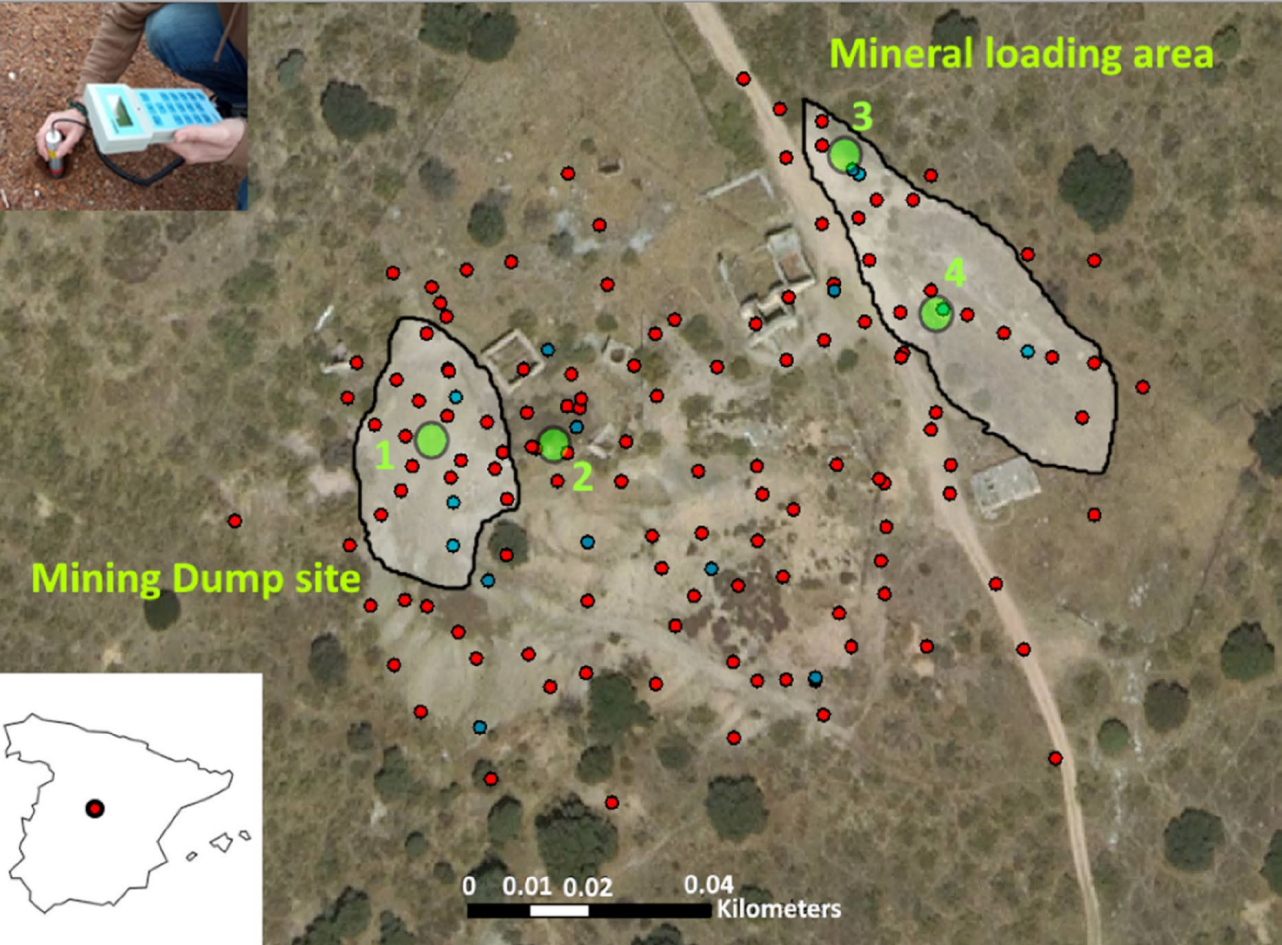
Location of the 141 points where measurements of ambient dose equivalent H*(10) (H*(10)_c_, H*(10)_C+S_, H*(10)_1 m_) and alpha (α_counts_), beta (β_counts_) and beta-gamma (β/γ_counts_) counts were made. The blue points indicate sampling locations for laboratory analysis. The four points shown in green correspond to the points where the models proposed by ArcMap 10.8.1 of ArcGIS and the proposed neural network (NN) were validated

The locations of the 141 points where measurements of dose rates and surface activities of α, β and β/γ were made are shown as red dots in Fig. 1. The blue dots represent the subset of points for which laboratory analyses were performed. The figure also shows the two most active areas of the mine, the ore loading area and the tailings dump. Samples were collected using a grid of approximately 20 cm × 20 cm and 5 cm deep. The points shown in green correspond to the four points measured in situ and which were used to validate the proposed models with the NN and with ArcMap 10.8.1 in ArcGIS.

### Measuring equipment and correlation of in-situ and laboratory measurements

#### In-situ measuring equipment

The equipment used for the in-situ radiological measurements was a LAMSE multi-probe monitor model MS6020 (Suárez et al., 2021). The probes used were: (i) CT115AB probe for measuring alpha (α_counts_) and beta (β_counts_) counts, (ii) CT115BG probe for measuring beta-gamma (β/γ_counts_) counts, and (iii) RD2L probe for measuring ambient dose equivalent H*(10). Spillover in the β counts was corrected by determining the percentage of alpha counts in the beta window using a plate with a known activity of ^241^Am. In addition, H*(10) values were measured in three different ways: (i) in contact (H*(10)_c_); (ii) in contact with a 5 cm lead shield (H*(10)_c+s_); and (iii) 1 m above the ground (H*(10)_1 m_).

#### Laboratory measuring equipment

The laboratory analyses included gross alpha and beta activity indices and gamma spectrometry measurements.

The method used to determine the gross alpha and gross beta activity consisted of forming an emulsion with a fixing agent, evaporating to dryness, and measuring in a continuous gas flow proportional counter (Lee et al., 2013). The gross alpha and beta activity indices were determined using a 50 mg aliquot of the dried sample at a constant weight. The aliquot was placed on a stainless steel plate with 8 grooves and a diameter of 5 cm. The sample was then fixed on the stainless steel plate by forming an emulsion by adding 0.5 mL of 33.3% (v/v) Tween20 solution. Finally, the Tween20 solution was evaporated to dryness under an infrared lamp and the residue obtained was dried in an oven at 180 °C to constant weight. The alpha and beta counts of the sample were determined using a low-background continuous gas flow proportional counter, Berthold model LB 770–2, for 1000 min.

The sample used for the gamma spectrometry measurement was prepared by drying at 105 °C to a constant weight and grinding a representative aliquot to a particle size of 250 μm. A 107 mL aliquot of the sample was placed in a cylindrical container with a diameter of 76 mm and a height of 30 mm (Suárez-Navarro et al., 2020). The cylindrical container was sealed to prevent loss of ^222^Rn, as the percentage of potential emission from this type of sample is between 10–20% (Sakoda et al., 2011). The samples were stored for 21 days to allow ^226^Ra to reach secular equilibrium with its short-lived gamma-emitting daughters ^214^Pb and ^214^Bi. The samples were measured using three different types of coaxial detectors: extended range (XtRa), reverse electrode (REGe) and broad energy range (BEGe). The relative efficiencies of the detectors were 115.7% (XtRa), 35.5% (REGe), and 48.0% (BEGe). The dimensions of the XtRa, REGe, and BEGe detectors were 84 mm, 58 mm, and 80 mm in diameter with lengths of 72 mm, 60 mm, and 30 mm, respectively. The thickness of the Fe/Pb shielding covering the detectors was 150 mm with a 3 mm Cu and Zn inner foil. The three detectors had a resolution of 2.0 keV for the 1.33 MeV photopeak of ^60^Co. The detectors were connected to a compact Mirion-Canberra DSA-LX electronics chain, which integrates the high voltage source, amplifier and analogue-to-digital converter. Efficiencies were calculated using LabSOCS software, and coincidence summing corrections were made using the Genie 2000 Peak-to-Total algorithm, which uses the total efficiency curve as a function of energy (Barba-Lobo et al., 2023). The spectra were acquired and analysed using the Genie 2000 Gamma Acquisition & Analysis software (CANBERRA, 2009). The spectral interferences of ^235^U at the 186 keV photopeak and of ^228^Ac at the 1460 keV photopeak were corrected using the algorithm developed in Suárez-Navarro et al. (Suárez-Navarro et al., ﻿^2018^). The photopeaks used in this study are shown in Table 1 (Be et al., 2016). The gamma spectrometry and gross alpha and beta activity indices are accredited by the National Accreditation Body (ENAC) according to the UNE-EN ISO/IEC 17025:2017 standard (UNE-Ie, 2017).

**Table 1:** Photopeaks used with their emission probabilities to determine the activity concentration of gamma emitters in the uranium, thorium and actinium series, together with ^40^ K and ^137^Cs

Natural radiactive series	Radionuclide	Energy (keV)	Photons per 100 disint
Uranium	^210^Pb	46.539 (1)	4.252 (40)
	^234Th^	63.20 (2)	3.75 (8)
	^226^Ra	186.211 (13)	3.555 (19)
	^214^Pb	351.932 (2)	35.60 (7)
	^214^Bi	609.312 (7)	45.49 (19)
		1120.287 (10)	14.91 (3)
		1764.494 (14)	15.31 (5)
	^234 mPa^	1001.441 (18)	0.856 (8)
Actinium	^235^U	143.761 (3)	10.94 (6)
		163.356 (3)	5.08 (3)
		185.720 (4)	57.0 (3)
		205.316 (4)	5.02 (3)
Thorium	^212^Pb	238.632 (2)	43.6 (5)
	^208^Tl	583.187 (6)	85.0 (3)
	^228^Ac	911.196 (6)	26.2 (8)
	^137^Cs	661.655 (3)	85.01 (20)
	^40^ K	1460.822 (6)	10.55 (11)

#### Relationships between *in-situ* and laboratory measurements

The equivalence between the in-situ radiological measurements and the laboratory analyses was verified through linear regression analysis using the 'stats' package from the statistical software RStudio version 2023.12.0.The linear relationships between the gross alpha and beta activity indices were determined directly by plotting the *in-situ* α_counts_ and β_counts_ against these indices. The ambient dose equivalent H*(10)_1 m_ and the gamma spectrometry measurements were verified using Eq. 1, which employs the coefficients from Saito et al. (Gil-Pacheco et al., 2020; Saito & Jacob, 1995).

1 $${\text{H}}^{*}{\left(10\right)}_{1\text{m}}=0.7\cdot \left[\left(5.7907\cdot {10}^{-3}\cdot {\text{C}}_{{234}_\text{Th}}+6.9\cdot {10}^{-5}\cdot \text{max}\left({\text{C}}_{{234}_\text{Th}}+{\text{C}}_{{226}_\text{Ra}}+{\text{C}}_{{210}_\text{Pb}}\right)+5.594\cdot {10}^{-2}\cdot {\text{C}}_{{226}_\text{Ra}}+4.0\cdot {10}^{-4}\cdot {\text{C}}_{{210}_\text{Pb}}\right)+\left(2.2145\cdot {10}^{-1}\cdot {\text{C}}_{{228}_\text{Ac}}+5.721\cdot {10}^{-2}\cdot {\text{C}}_{{212}_\text{Pb}}+3.26\cdot {10}^{-1}\cdot {\text{C}}_{{208}_\text{Tl}}\right)+\left(4.17\cdot {10}^{-2}\cdot {\text{C}}_{{40}_\text{K}}\right)+\left(1.24\cdot {10}^{-1}\cdot {\text{C}}_{{137}_\text{Cs}}\right)\right]$$

where ${\text{C}}_{{234}_\text{Th}}$, ${\text{C}}_{{226}_\text{Ra}}$, ${\text{C}}_{{210}_\text{Pb}}$, ${\text{C}}_{{228}_\text{Ac}}$, ${\text{C}}_{{212}_\text{Pb}}$, ${\text{C}}_{{208}_\text{Tl}}$, ${\text{C}}_{{40}_\text{K}}$ and ${\text{C}}_{{137}_\text{Cs}}$ are the activity concentrations of ^234^Th, ^226^Ra, ^210^Pb, ^228^Ac, ^212^Pb, ^208^Tl, ^40^K and ^137^Cs, respectively, in Bq kg^−1^ and 0.7 is the factor to transform Gy into Sv (Expósito-Suárez et al., 2023). The secular equilibrium between ^234^Th and ^238^U (T_1/2_ = 24.10d) was tested by measuring 10% of the samples in two periods 90 days apart and checking that the activity concentrations obtained were statistically equal.

### Geostatistical modelling with ArcGIS (Co-Kriging) and prediction of in-situ values and activity concentrations of radionuclides from natural radioactive series

**Fig. 2:**
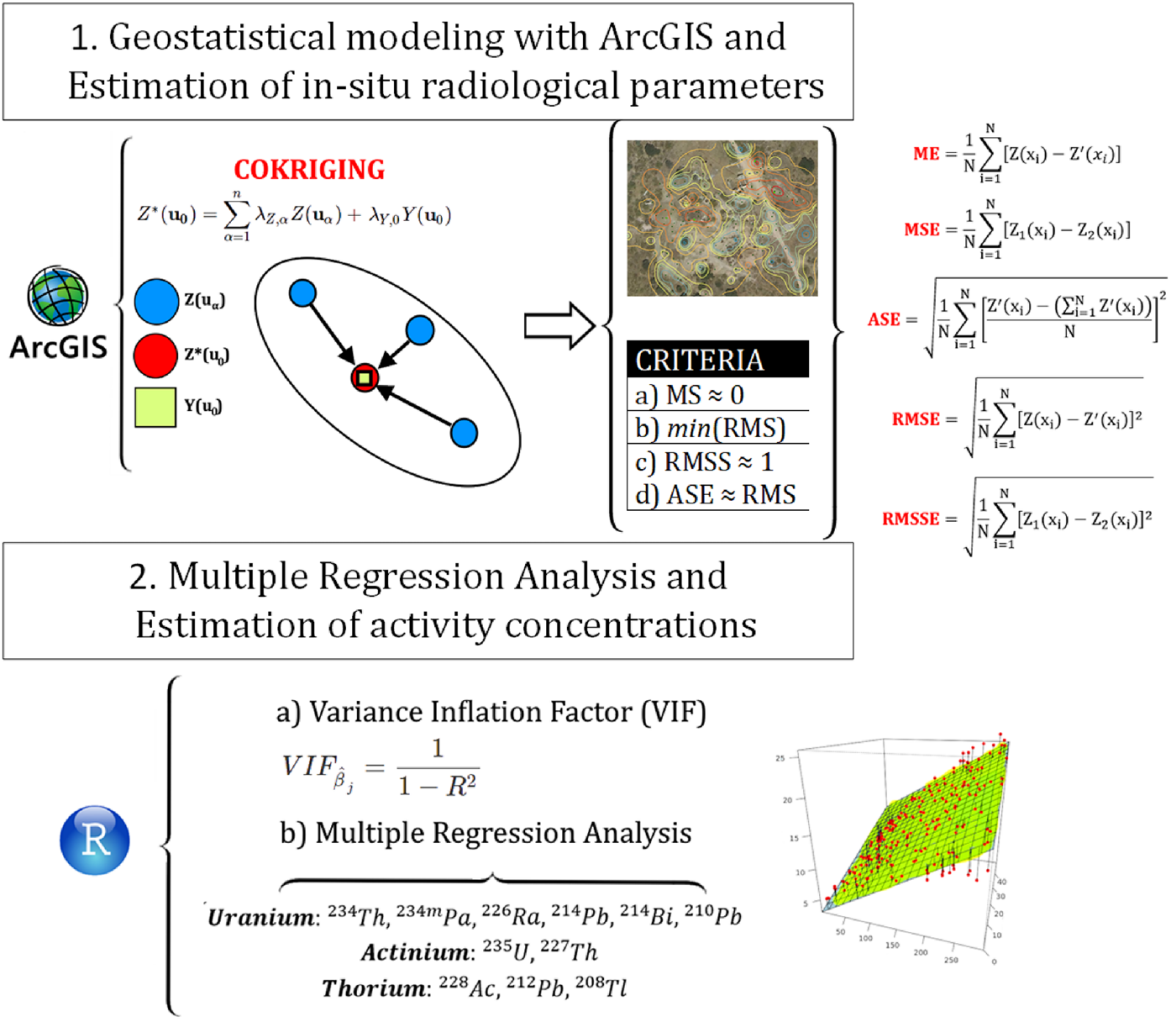
Geostatistical modelling with Co-Kriging and prediction of *in-situ* values and activity concentrations of radionuclides from natural radioactive series. In the first stage the radiological parameters were estimated using these spatial autocorrelation of their geographical coordinates through the semivariogram. The different cokriging models were compared using the mean standardised error (ME), average standard error (ASE), root mean square error (RMSE), mean standard error (MSE) and root mean square standardised error (RMSSE). The parameters of these expressions are: Z(x_i_) is the measured value; Z'(x_i_) is the estimated value; and Z_1_(x_i_) and Z_2_(x_i_) are the standardised values. In the second step, the activity concentrations of different natural radionuclides were estimated with RStudio from the radiological parameters using multiple regression analysis. VIF represents the variance inflation factor and R^2^ is the coefficient of determination

Figure 2 depicts an outline of the process used to geostatistically model the 141 sampling points. It also shows the statistical procedure used both to determine the *in-situ* radiological parameters from the geographical coordinates and to estimate the activity concentration of radionuclides from natural radioactive series based on the *in-situ* radiological parameters.

#### Geostatistical modelling with Co-Kriging (CK) from ArcGIS

The 141 sampled points were input into a geographic information system (ArcGIS 10.8.1) using their geographic coordinates. The geostatistical method chosen for interpolation was CK, which has been successfully used in previous studies (Caro et al., 2013; Gil-Pacheco et al., 2020).

The kriging technique weights the experimental values around the point to be predicted, taking into account both the distance between the measured points and the spatial relationships existing between them, quantified by autocorrelation. The value obtained is an unbiased linear estimator, with minimal estimation error and no bias due to the conditions imposed in the development of the kriging equations (Childs, 2004). The optimal estimation achieved through kriging occurs when the distribution of observations is normal. However, this condition is not always met in natural phenomena. The first step was therefore to check whether the six experimental value distributions (H*(10)_c_, H*(10)_c+s_, H*(10)_1 m_, α_counts_, β_counts_ and β/γ_counts_) followed a normal distribution. Normality was assessed using the standardised kurtosis value (ranging between ± 2) and the difference between the arithmetic mean and the median of less than 1%. Data from distributions that did not meet these conditions were logarithmically transformed.

The geostatistical analysis of multiple variables was conducted using the CK method, which, in addition to the spatial correlation of the main variable, utilises spatial information from secondary variables to enhance the prediction surface. The normal or log-normal distributions obtained were modelled using ordinary and simple CK with sets of 4, 3, and 2 radiological values. The different sets were validated by sequentially omitting a point in the dataset and predicting a value for that point using the rest of the data. The measured values were subsequently compared with the predicted values to obtain the prediction error, allowing the best models to be selected. Cross-validation was performed using the following parameters: (i) mean standardised error (ME); (ii) mean standard error (MSE); (iii) average standard error (ASE); (iv) root-mean-square error (RMSE); and (v) root-mean-squared standardised error (RMSSE). The models selected from among those proposed were those that met the following conditions (Yang et al., 2009):

1. ME ≈ 0, indicating unbiased predictions;
2. low MSE and RMSE values, close to 0, indicating that the predictions did not deviate significantly from the experimental values;
3. RMSSE ≈ 1, showing low dispersion of model standard errors;
4. ASE ≈ RMSE, indicating that the variability of the predictions was adequately assessed.

#### Estimation of radionuclide activity concentrations of natural radioactive series from radiological parameters measured in situ

The activity concentrations of radionuclides from natural radioactive series were determined using various radiological parameters: H*(10)_c_, H*(10)_c+s_, H*(10)_1 m_, α_counts_, β_counts_ and β/γ_counts_. The models were obtained from multiple regression analysis (MRA) using the statistical tool RStudio and the 'stats' and 'car' packages, version 2023.12.0. Collinear radiological parameters were eliminated to accurately assess the influence of each variable in the proposed models. Collinearity was determined using the variance inflation factor (VIF). In addition, parameters that were not collinear but which did not reach the required level of statistical significance in the MRA were also excluded from each of the adjusted models (Cano et al., 2023).

### NN architecture used to fit experimental values and obtain predictions of radiometric values and activity concentrations of radionuclides from natural radioactive series

The two NNs used in this study enabled two different tasks (Fig. 2): (i) the establishment of complex non-linear relationships between the variables measured in situ (H*(10) and α, β and β/γ surface activity) with geographical coordinates; and (ii) the prediction of the activity concentration of radionuclides belonging to the natural radioactive series of uranium, actinium and thorium, based on the radiometric measurements taken in situ. By optimising both numerical methods, it is possible to move from discrete fields of variables to continuous models. Numerical methods based on Artificial Intelligence (Partin et al., 2023; Vapnik, 2013) were employed using the Python programming language within development frameworks like GoogleColab. Two fundamental machine-learning modules were utilized:

(i) The sklearn.cluster module provides tools for data clustering, including DBSCAN (Density-Based Spatial Clustering of Applications with Noise);
(ii) Keras and TensorFlow libraries were used to optimize convolutional neural networks fully connected neural networks and binary classification models, which form the basis of Garson's algorithm.

#### Fitting and estimating of *in-situ* measurements based on GPS coordinates using the AE algorithm

The first process necessary for predicting radiological measurements from geographical coordinates was the standardisation of the experimental data. This process was essential due to the highly heterogenous values obtained in situ, as the study area was a disturbed dump. In addition, the topography of the dump site prevented the creation of a network of equidistant points, as access to some areas was unsafe. This standardisation process was carried out by optimising an autoencoder (AE) algorithm (Benavente & Correcher, 2023). As independent variables, the AE algorithm used a data matrix of 8 and 141 observations corresponding to each sampling point. These independent variables were M^i^ = [^i^H*(10)_c_, ^i^H*(10)_c+s_, ^i^H*(10)_1 m_, ^i^α_counts_, ^i^β_counts_, ^i^γ/β_counts_] together with geographical coordinates [^i^Latitude, ^i^Longitude] for each observation. The algorithm performs an initial coding stage followed by a decoding stage. The encoding is performed starting from a convolutional stage consisting of 32 filters that generate 32 × 8 data sets. The output of each neuron (${\text{Z}}_{\text{n},\text{j}}$) is represented by Expression 2.

2 $${{\text{Z}}_{\text{n},\text{j}}=\left.{\phantom{i}}_{\phantom{i}}{\phantom{i}}^{\text{n}}{\text{F}}_{\text{j}}\left(\sum_{\text{i}=\text{j}}^{\text{j}+\text{Kernel Size}}{\phantom{i}}_{\phantom{i}}{\phantom{i}}^{\text{n}}{\upomega }_{\text{ij}}^{\phantom{i}}\cdot {\text{M}}^{\text{i}}+{\phantom{i}}_{\phantom{i}}{\phantom{i}}^{\text{n}}{\text{b}}_{\text{j}}\right)\right|}_{\forall \text{ n }\in \left[\text{1,32}\right];\,\forall \,\text{ j }\in \left[\text{1,7}\right]}$$

where ^n^F_j_ is an activation function RELU and ${\phantom{i}}^{\text{n}}{\upomega }_{\text{ij}}$ represents the weights of the synaptic relationships between the neurons. The second convolutional stage was designed with a single filter generating two-dimensional vectors represented by Expression 3.

3 $${{\text{K}}_{\text{l}}={\phantom{i}}_{\phantom{i}}{\phantom{i}}^{\text{l}}\text{G}\left[{\left.\sum_{\text{j}=1}^{32}\left(\sum_{\text{d}=1}^{7}{{\phantom{i}}_{\phantom{i}}{\phantom{i}}^{\text{d}}\upomega }_{\text{j}}\cdot {\text{Z}}_{\text{d},\text{k}}\right)+{\phantom{i}}_{\phantom{i}}{\phantom{i}}^{\text{l}}{\text{b}}_{\phantom{i}}\right|}_{\forall \text{ l }\in \left[\text{A},\text{B}\right]}\right]}$$

where ^l^G is a RELU activation function, and the weights in this case are represented by ${\phantom{i}}_{\phantom{i}}{\phantom{i}}^{\text{d}}{\upomega }_{\text{j}}$. The weights of the synaptic relationships between neurons ${\phantom{i}}{\phantom{i}}^{\text{n}}{\upomega }_{\text{ij}}$ and ${\phantom{i}}_{\phantom{i}}{\phantom{i}}^{\text{d}}{\upomega }_{\text{j}}$ are optimised during the training phase by minimising the Loss Function (LF) using Expression 4.

4 $$\text{LF}=\sum_{\text{k}=1}^{141}\sum_{\text{i}=1}^{8}\frac{{\left({{\phantom{i}}_{\phantom{i}}{\phantom{i}}^{(\text{k})}\text{M}}_{\text{i}}-{\phantom{i}}_{\phantom{i}}{\phantom{i}}^{\left(\text{k}\right)}{\text{S}}_{\text{i}}\right)}^{2}}{2}$$

The minimisation process was performed using the iterative method known as gradient descent, which is defined by Expression 5.

5 $${{\phantom{i}}{\phantom{i}}^{\left(\text{n}\right)}{\text{w}}_{\text{ij}}}_{\left[\text{t}+1\right]}={{\phantom{i}}{\phantom{i}}^{\left(\text{n}\right)}{\text{w}}_{\text{ij}}}_{\left[\text{t}\right]}-\alpha \cdot {\left.\frac{\partial \left(\text{LF}\right)}{\partial {\phantom{i}}{\phantom{i}}^{\left(\text{n}\right)}{\text{w}}_{\text{ij}}}\right|}_{\left[\text{t}\right]}$$

#### Density-based spatial clustering of applications with noise (DBSCAN)

The density-based spatial clustering of applications with noise (DBSCAN) algorithm is a numerical method of unsupervised learning capable of discriminating areas with a high density of points (Suárez-Navarro et al., 2024), compared to others with a low density.

This method is based on identifying core points, which are primarily characterised by two parameters. The first is the minimum number of points (referred to as "MinPts") in their neighbourhood, which includes the point itself. The second parameter is the neighbourhood (denoted as ε), or the area where the Euclidean distance (expressed by Expression 6) between the potential core point and other points in the set, according to Expression 6, is less than a certain ε value.

6 $${\upvarepsilon }_{\text{ij}}=\sqrt{{\left({\text{A}}_{\text{i}}-{\text{A}}_{\text{j}}\right)}^{2}+{\left({\text{B}}_{\text{i}}-{\text{B}}_{\text{j}}\right)}^{2}}$$

where A and B are the variables of the vector in the bottleneck region of the method.

When all the nodes are connected, either directly or indirectly, clusters are formed. In our problem, MinPts and ε were set to 3 and 0.125 respectively. The algorithm identified 2 clusters or data sets (referred to as Zone 0 and 1), in addition to Zone -1, which contains the points that the method was unable to group.

#### Prediction of radiological values from geographic coordinates

The estimation of *in-situ* measurements from geographic coordinates [^i^Latitude, ^i^Longitude] was performed through linear interpolation using values obtained from clusters with the DBSCAN algorithm. The radiological values were determined by the matrix M_i_ = [^i^H*(10)_C_, ^i^H*(10)_C+S_, ^i^H*(10)_1 m_, ^i^α_Counts_, ^i^β_Counts_, ^i^γ/β_Counts_]. Estimated radiological values were determined by weighted averaging between the distance of the interpolated point (^(k)^d_Min_) and the nearest of the 3 clusters or zones identified by the DBSCAN algorithm, as expressed in Eq. (7).

7 $${\text{M}}_{\text{i}}\left({x}_{i},{y}_{i}\right)=\frac{\frac{{\phantom{i}}{\phantom{i}}^{(0)}P\left({x}_{i},{y}_{i}\right)}{{\phantom{i}}{\phantom{i}}^{(0)}{d}_{Min}}+\frac{{\phantom{i}}{\phantom{i}}^{(1)}P\left({x}_{i},{y}_{i}\right)}{{\phantom{i}}{\phantom{i}}^{(1)}{d}_{Min}}+\frac{{\phantom{i}}{\phantom{i}}^{(-1)}P\left({x}_{i},{y}_{i}\right)}{{\phantom{i}}{\phantom{i}}^{(-1)}{d}_{Min}}}{\frac{1}{{\phantom{i}}{\phantom{i}}^{(0)}{d}_{Min}}+\frac{1}{{\phantom{i}}{\phantom{i}}^{(1)}{d}_{Min}}+\frac{1}{{\phantom{i}}{\phantom{i}}^{(-1)}{d}_{Min}}}$$

where ${\phantom{i}}{\phantom{i}}^{(k)}P\left({x}_{i},{y}_{i}\right)$ represents the interpolation polynomials. The different zones were represented by surface plots as a function of the coordinates.

#### Prediction of activity concentrations of radionuclides from natural radioactive series based on *in-situ* radiological values

**Fig. 3:**
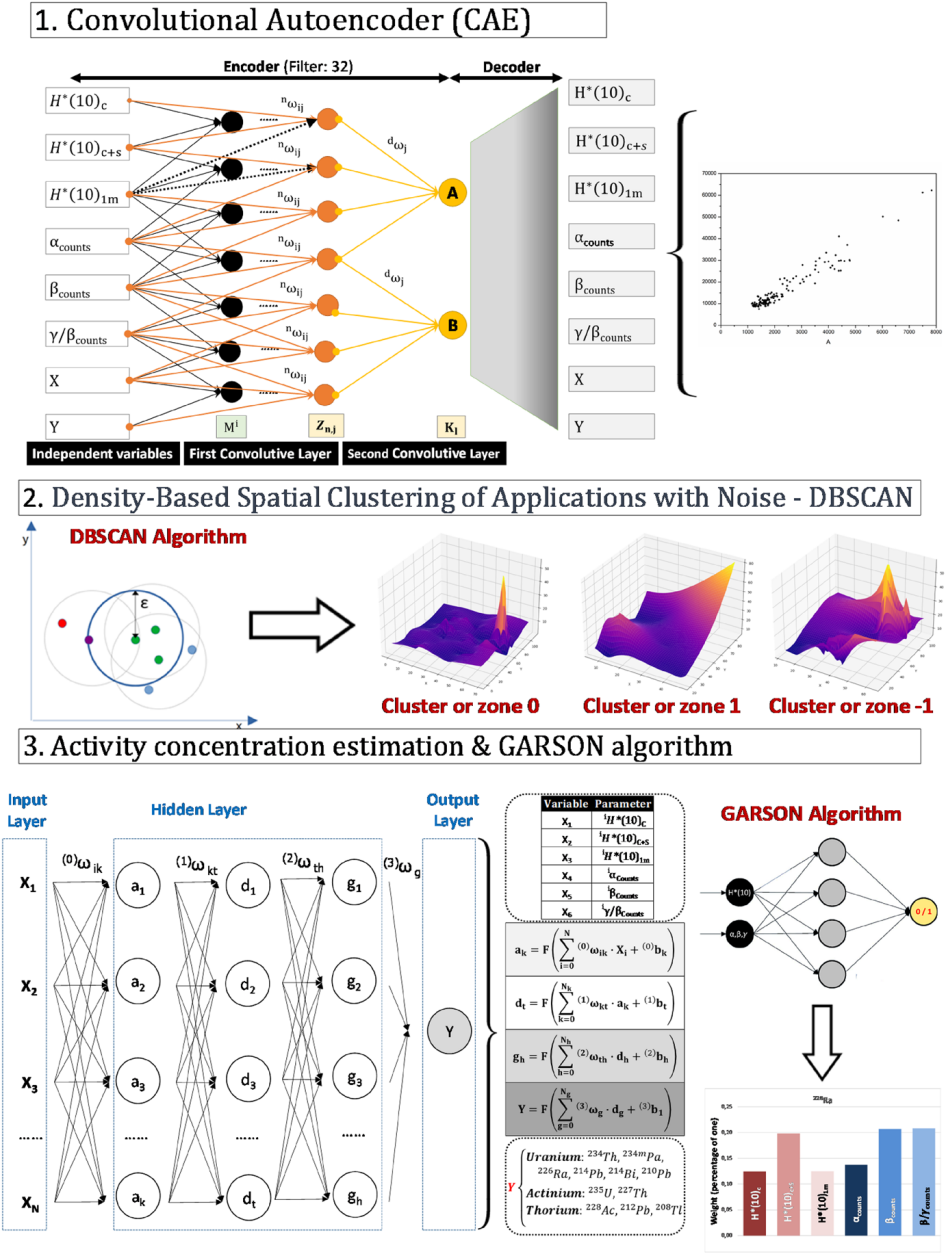
Algorithms and NNs used in this study: (1) convolutional autoencoder (CAE) algorithm used for experimental point classification; (2) density-based spatial clustering of applications with noise (DBSCAN) algorithm; and (3) fully connected NN and Garson algorithm for estimating activity concentrations of radionuclides from natural radioactive series

The activity concentrations of radionuclides from the three natural radioactive series were estimated using 9 different fully connected NNs, whose architecture is shown in Fig. 3 (Romero-Salido et al., 2023). The NNs were constructed individually for each radionuclide to investigate the influence of different *in-situ* radiological parameters. The input layer of each NN consisted of the *in-situ* radiological data: ^i^H*(10)_c_, ^i^H*(10)_c+s_, ^i^H*(10)_1 m_, ^i^α_counts_, ^i^β_counts_, ^i^γ/β_counts_, represented in Fig. 2 as X_1_–X_6_. The hidden layer activation functions (a_k_, d_t_ and g_h_) were of the RELU type to speed up convergence. However, the activation function of the output layer was of the sigmoid type because the input values were normalised between 0 and 1. The weights of the synaptic relations (^(k)^ω_ij_) were optimised by minimising the loss function (LF) given by Expression 8.

8 $$\text{LF}=\sum_{\text{k}=1}^{141}\frac{{\left({{\phantom{i}}_{\phantom{i}}{\phantom{i}}^{(\text{k})}\text{C}}_{\text{i}}-{\phantom{i}}_{\phantom{i}}{\phantom{i}}^{\left(\text{k}\right)}{\text{Y}}_{\text{i}}\right)}^{2}}{2}$$

where C_i_ (Bq kg^−1^) is the concentration of the activity of the radionuclide studied, estimated from the *in-situ* radiological values of the k sampled points.

The Garson algorithm was used to study the weight of the different *in-situ* radiological parameters that made up the input layer (independent variables). The Garson algorithm consists of a supervised binary NN classification, the result of which is the percentage (expressed as a ratio) of each of the independent variables (Maozhun, 2017). The binary values were established based on the criterion of being either above or below the mean activity concentration C_i_ of a specific radionuclide (0 or 1 respectively).

### Comparison and validation of the results from the neural network with those obtained through Co-Kriging (CK) and multiple regression analysis

Different models made with CK from ArcGIS and the NN were compared using the mean standardised error (ME), average standard error (ASE), root-mean-squared error (RMSE), mean standard error (MSE), and root-mean-squared standardised error (RMSSE)(Yang et al., 2009). The expressions for these parameters are shown in Fig. 2. The calculated parameters differ from those described in Sect.  2.3.1 because cross validation obtains the parameters by sequentially omitting a point in the dataset and predicting the value for that point using the rest of the data. ArcGIS then compares the measured values with those predicted by the model to determine the prediction error. However, the comparison between CK values and NN was made using the MSE parameter, which was obtained by standardising the estimated values with respect to the mean. In addition, the values of the RMSE and RMSSE parameters were obtained by standardising the estimated values with respect to the standard deviation.

The validation of the results obtained from the NNs and through CK and MRA was carried out at 4 sampling points four months after the initial sampling. The validation points are shown in green in Fig. 1. The 4 points were reprocessed following the procedures described in Sect. "Measuring equipment and correlation of in-situ and laboratory measurements". However, in this case the gross alpha and beta activity indices were not determined as these were not necessary to validate the results. The individual comparison of the experimental values and those obtained by ArcGIS plus MRA and NNs was based on the relative bias (RB%). In addition, the accuracy and precision of the total set of predicted values at the 4 sampled points were tested using the Student's t-test and Fisher's F-test, respectively. Both statistical tests were performed with 2 tails and an α of 0.05 to test for significant differences between the means and variances of the different outcomes. In addition, the Student's t-test was used, depending on whether the variances were equal (homoscedastic) or not (heteroscedastic) (Miller & Miller, 2018).

## Results

### Comparison of in-situ radiological parameters and laboratory measurements

**Fig. 4:**
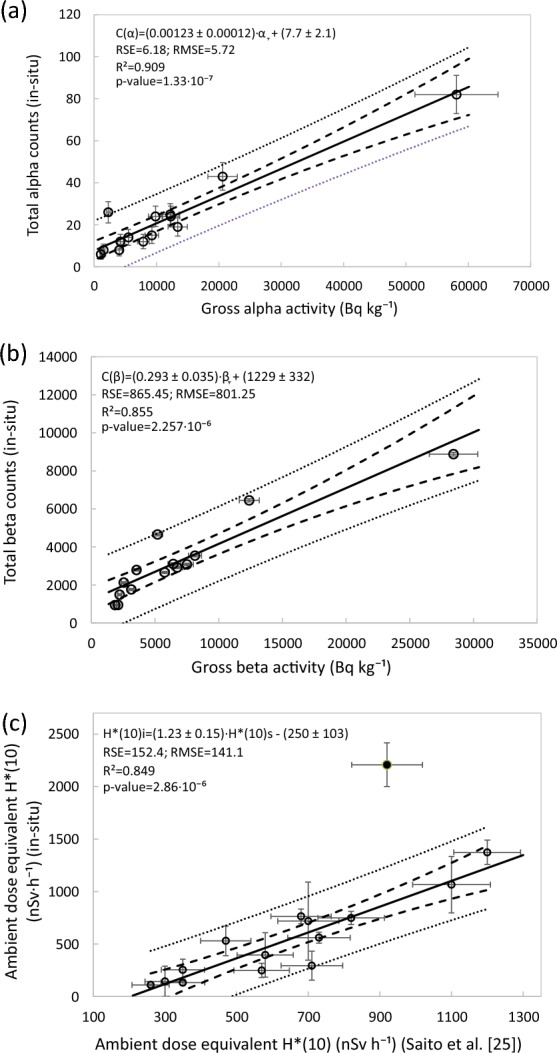
Comparison of *in-situ* radiological parameters measured with the LAMSE MS6020 multi-sensor instrument as a function of laboratory measurements: (1) total counts measured with the CT115BG probe as a function of the gross alpha activity index in solid samples; (2) total counts measured with the CT115BG probe as a function of the gross beta activity index in solid samples; and (3) environmental dose rate H*(10) measured with the RD2L probe as a function of the theoretical ambient dose equivalent H*(10) calculated using the parameters of Saito et al. and gamma spectrometry measurements. The bar line corresponds to the confidence interval, while the dotted line corresponds to the prediction interval

Figure 4 shows the three comparisons made between the *in-situ* radiological parameters (α_counts_, β_counts_, H*(10)_1 m_) recorded with the LAMSE MS6020 portable multi-sensor monitor and the laboratory parameters (gross alpha activity indices, gross beta activity, and gamma spectroscopy with HPGe detector). All three fits showed that there was a statistically significant relationship at the 95% significance level between the *in-situ* parameters and the laboratory measurements, as the *p* values were less than 0.05. The linear fit models correctly represented 84.9% of the dispersion of the independent variables. Furthermore, the activity index values and the α_counts_ produced the best fit, as the RSE value was closest to 0. In addition, this relationship obtained a lower fitting error than in the case of the gross beta activity index and gamma spectroscopy, as the RMSE value was the lowest. On the other hand, the relationship between gamma spectroscopy and H*(10) gave an anomalous value that was rejected from the study.

### Estimation of radiological parameters based on geographical coordinates estimated by Co-Kriging (CK) and by neural network (NN)

**Fig. 5:**
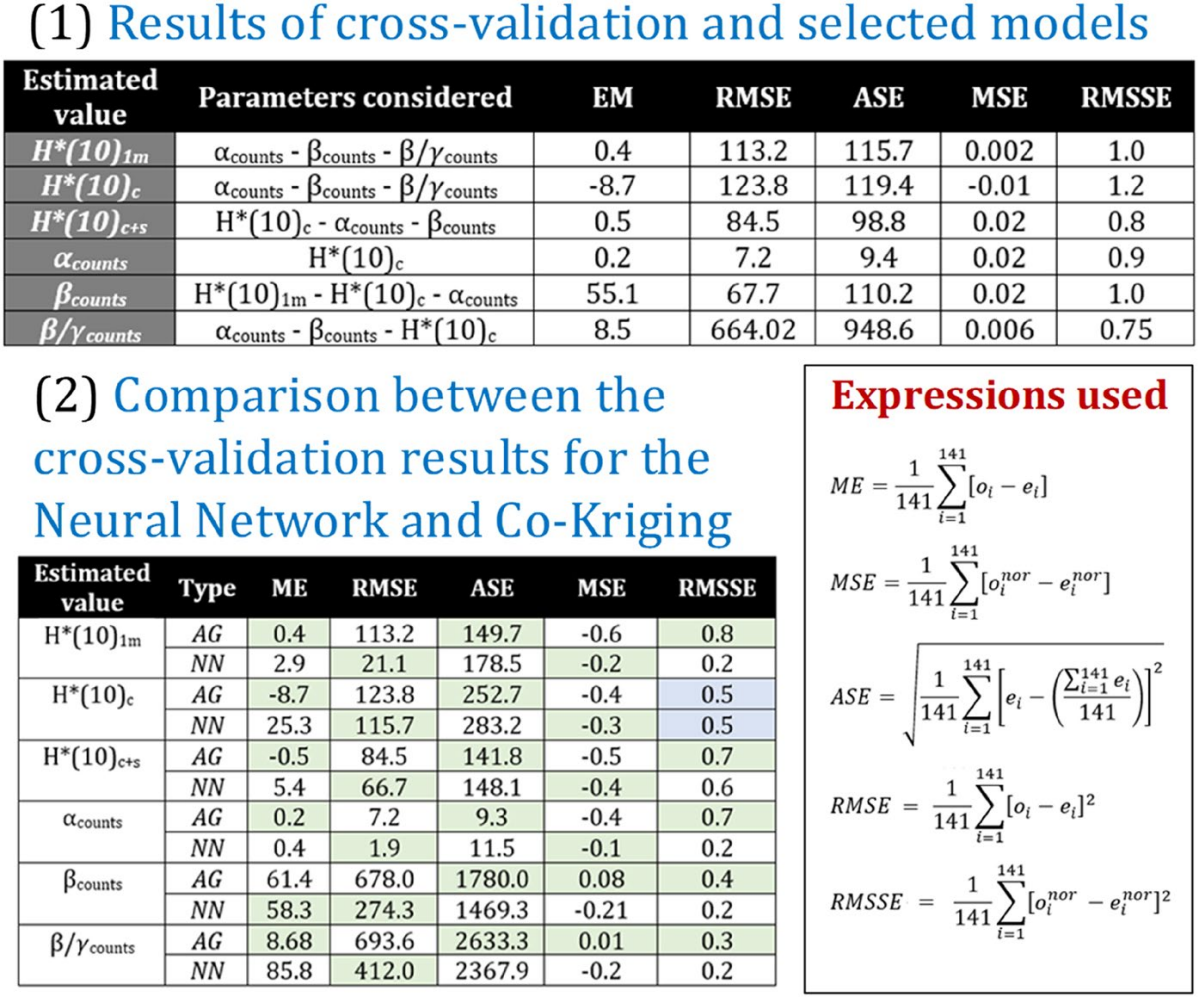
Comparison of cross-validation results: (1) results obtained for the 6 ArcGIS models; and (2) comparison between the Co-Kriging (CK) and neural network (NN) models used to estimate H*(10)_1 m_, H*(10)_c_, H*(10)_c+s_, α_counts_, β_counts_ and β/γ_counts_. The expressions used in the calculation are also shown, where ${o}_{i}$ is the observed value in the sample, ${e}_{i}$ is the value estimated by the model, and ${o}_{i}^{nor}$ and ${e}_{i}^{nor}$ are the same values but standardised to the standard deviation

Figure 5 shows the best models proposed by ArcGIS and the comparison between the predictions obtained by CK and the NN. All models followed a normal distribution except for the H*(10)_c_ model to which a logarithmic transformation was applied to obtain a better estimate of the values. The statistical parameters used in the cross-validation between CK and the NN were the same as those used to select the models provided by CK. However, as discussed in Sect. "Comparison and validation of the results from the neural network with those obtained through Co-Kriging (CK) and multiple regression analysis", the values of ASE, MSE and RMSSE obtained were different from those obtained by ArcGIS due to the way in which the standardisation of the estimated values was performed. The values predicted by NN and ArcGIS were equivalent and met the criteria set out in Sect.  Geostatistical modelling with Co-Kriging (CK) from ArcGIS. However, the predictions of β_counts_ and β/γ_counts_ showed a poorer assessment of variability in both CK and NN, as the RMSE and ASE values were significantly different.

**Fig. 6:**
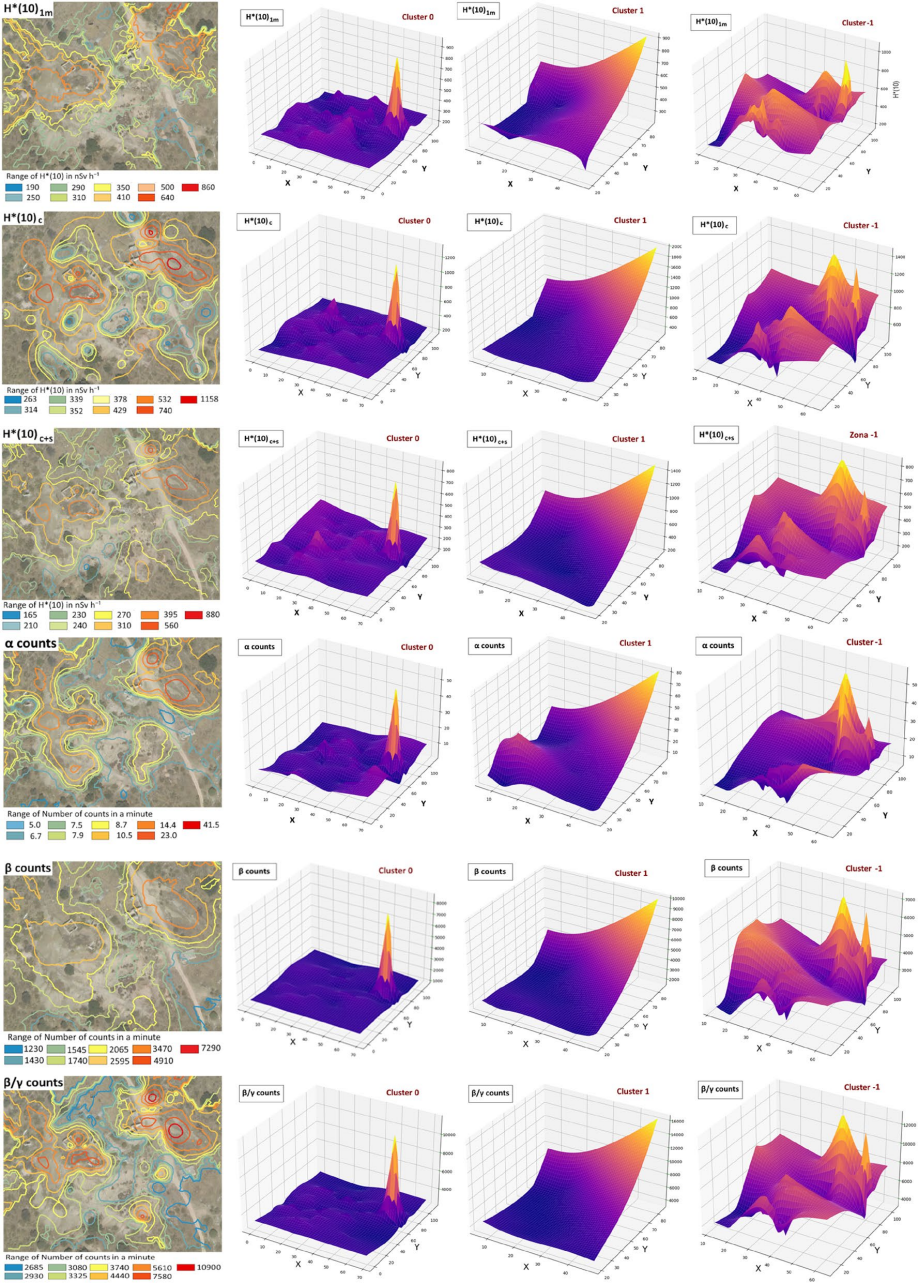
ArcGIS maps and surface plots of the 3 clusters obtained with the DBSCAN algorithm, where cluster 0 corresponds to the grouping of 58.86% of the experimental values, while clusters 1 and -1 correspond to 8.51% and 32.64%, respectively. Both ArcGIS and NN clearly delineate the two areas with higher radioactivity, namely the tailings and the mineral loading area

Figure 6 shows the maps obtained using ArcGIS and the surface plots of the three clusters obtained using the DBSCAN algorithm for each of the radiological parameters. Both methods show two areas of higher dose and surface contamination corresponding to the tailings and the area where the Cu mineral was loaded. The surface plots show the following percentages of experimental values clustered in the study area: cluster 0 with 58.86%; cluster 1 with 8.51%; and cluster -1 with 32.64%. Cluster -1 represents those experimental points that the DBSCAN model was unable to group into zones of at least 3 points. This cluster clearly highlights the areas of higher radioactivity, where it is logical to expect greater heterogeneity in the measurements of H*(10) and the α, β and β/γ counts.

### Comparison between the activity concentrations of radionuclides from natural radioactive series obtained using the NN and multiple regression analysis (MRA)

**Fig. 7:**
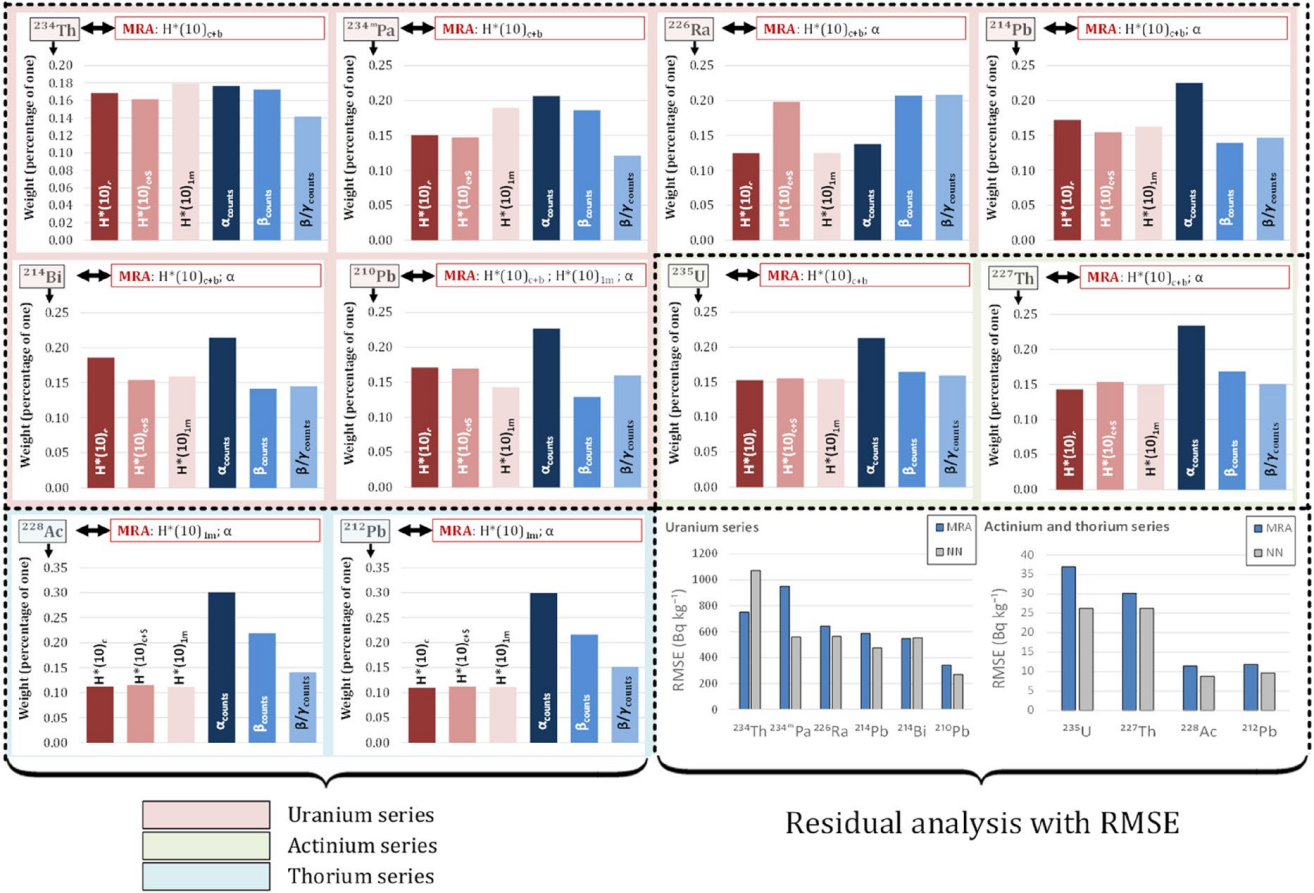
Parameters obtained through multiple regression analysis and the weights of the parameters obtained using the Garson algorithm, along with a comparison of prediction errors obtained using the root mean standardised error (RMSE)

The activity concentrations estimated by multiple regression analysis (MRA) and neural network (NN) were derived from *in-situ* radiological data and laboratory gamma spectrometry results obtained from 24 samples (17% of the total *in-situ* measurements). The MRA allowed a prediction model to be obtained using parameters that did not show collinearity and had a statistically significant goodness of fit, i.e., with a p-value of less than 0.05. The parameters that met these two conditions are shown in the graphs of Fig. 7 for each radionuclide within a red box. On the other hand, the bar graphs show the weights obtained for the *in-situ* radiological data of each radionuclide using the Garson algorithm for the NN. The most influential parameters according to the MRA for the uranium radiometric series were H*(10)_c+s_, H*(10)_1 m_ and the α counts. The NN distributed the weight of the factors more evenly in its predictions, although it also gave greater weight to the α counts of all radionuclides except ^226^Ra. The predictions for the actinium series again showed a higher weight of α counts compared to other parameters, both for the MRA and NN. The MRA again showed a greater dependence of its predictions on H*(10)_c+s_, while in the NN the weight was spread over all the other parameters. Finally, the predictions for the thorium radioactive series showed a dependence on H*(10)_1 m_ and α counts for the MRA, while the NN gave higher weights to surface activity values (α_counts_, β_counts_ and β/γ_counts_). The predictions obtained using the MRA and NN were compared on the basis of the RMSE value. The bar chart shows, principally, that the RMSE values are lower in the case of the NN compared to the MRA, indicating better activity concentration predictions as the model error is lower.

### Validation of the predictions obtained at the 4 verification points

**Fig. 8:**
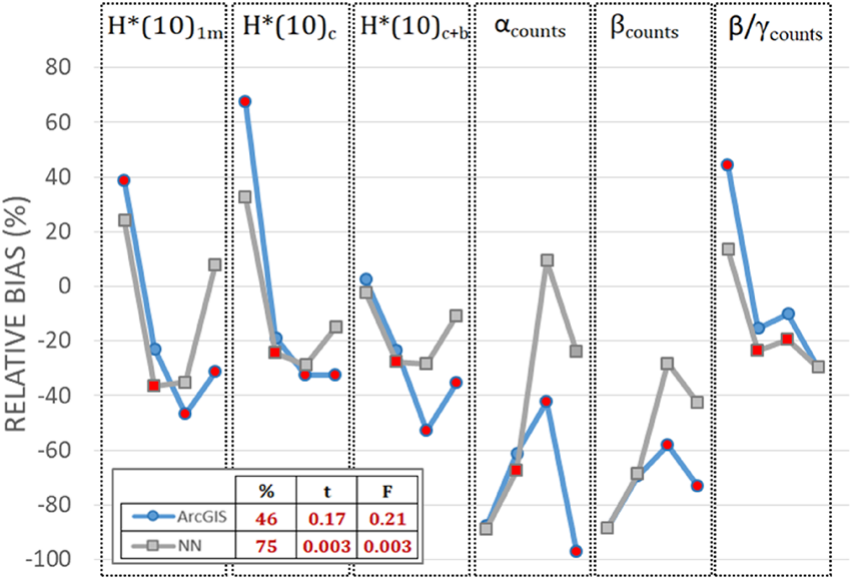
Validation of radiological parameters (H*(10)_c_, H*(10)_c+s_, H*(10)_1 m_, α_counts_, β_counts_ and β/γ_counts_) estimated from the CK and the NNs in 4 verification samples. The points shown in red correspond to the values most different from the experimental value. The data used to generate these graphs are shown in Figure S.1 in the supplementary information

**Fig. 9:**
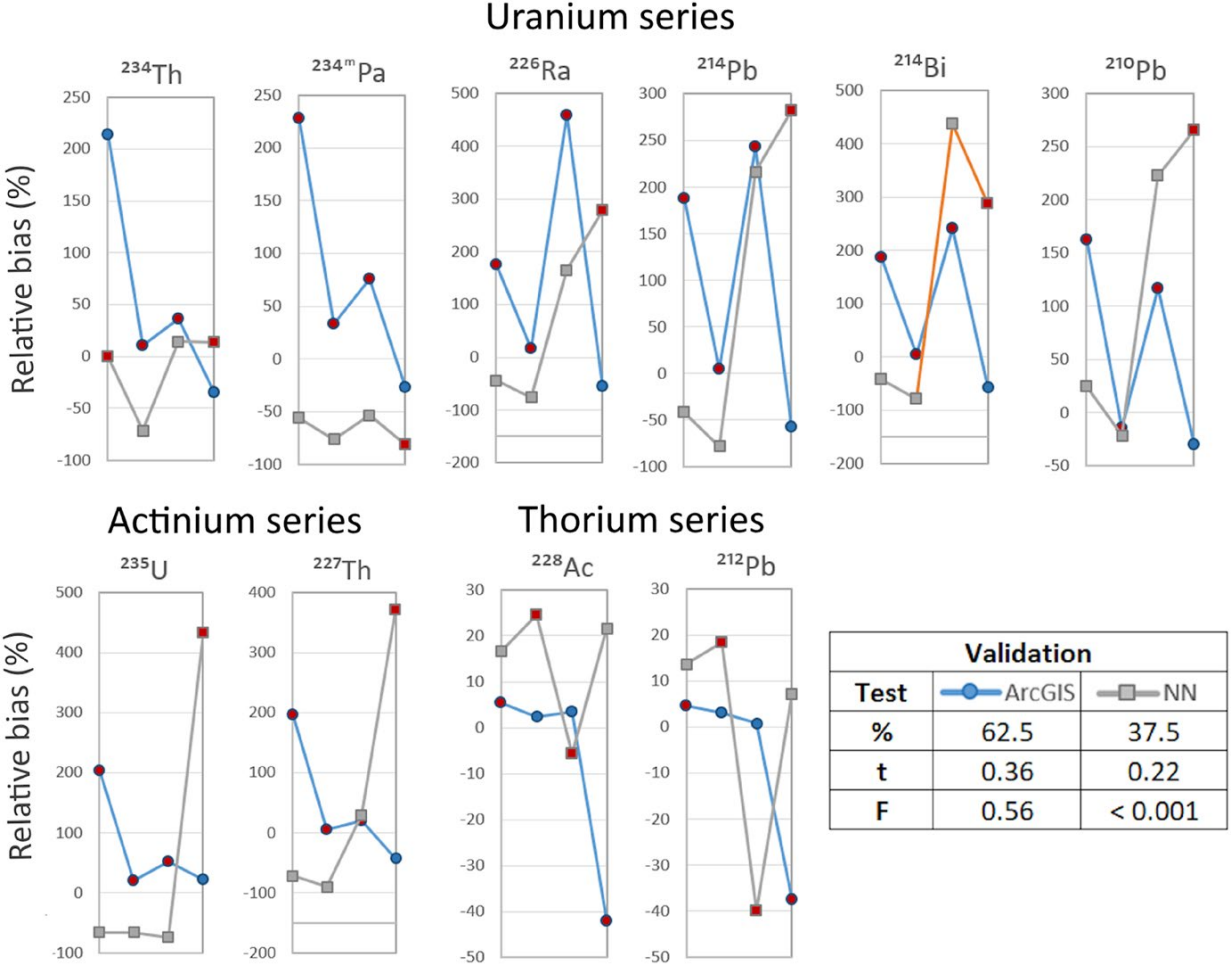
Validation of the activity concentrations of the natural radioactive series of uranium (^234^Th, ^234m^Pa , ^226^Ra, ^214^Pb, ^214^Bi and ^210^Pb), actinium (^235^U and ^231^Th) and thorium (^228^Ac and ^212^Pb) estimated from the radiological parameters (H*(10)_c_, H*(10)_c+s_, H*(10)_1 m_, α_counts_, β_counts_, and β/γ counts) in ArcGIS (CK), using the MRA and NN on four verification samples. The points shown in red correspond to the values most different from the experimental value. The data used to generate these graphs are shown in Figure S.1 in the supplementary information

Figures 8 and 9 show the estimates obtained for the 4 control points using the CK and the NN for the *in-situ* radiological data (H*(10)_c_, H*(10)_c+s_, H*(10)_1 m_, α_counts_, β_counts_ and β/γ_counts_) and for the MRA and the NN with respect to the activity concentrations obtained through gamma spectrometry. Both CK and the NN overestimated the radiological data based on the coordinates at the 4 control points. The percentage relative differences (RB%) between the predicted and experimental values were lower for the NN (74%) compared to ArcGIS (46%). The variances obtained between the predicted and experimental values were less than 0.05 for both CK and the NN, reflecting the positive bias observed in the RB%. However, the differences between the means of the experimental and predicted values were statistically comparable for both CK (*p* = 0.17) and NN (*p* = 0.21).

The activity concentrations obtained with the NN were less accurate than those obtained by the MRA, as the *p* value obtained for the F-test was less than 0.05 (Fig. 9). However, the means were statistically comparable for both the NN and MRA. When comparing the individual relative biases, the MRA achieved the lowest differences in 62.5% of cases.

## Discussion

The results obtained confirm our hypothesis that a NN produces results equivalent to CK together with a MRA, both for radiological measurements (ambient equivalent dose and surface activity) obtained from spatial coordinates, and when estimating the activity concentrations of radionuclides belonging to the 3 natural radioactive series. The first step in confirming the hypothesis was to verify that the *in-situ* measurements were statistically comparable to the laboratory measurements. This verification was carried out using linear regressions. The three parameters tested in the field, namely α_counts_, β_counts_ and H*(10)_1 m_, were statistically comparable with the gross alpha and gross beta activity indices and with gamma spectrometry, as the p-values obtained in all three regressions were less than 0.05. The variation was explained by 85% in all three models, as indicated by the R^2^ coefficients. There was only one outlier, which was rejected in the case of H*(10). The measurements taken in the study area are therefore reproducible with respect to those made in the laboratory.

The main drawback of the study area was the heterogeneity of the measurements taken and the impossibility of creating an evenly spaced point grid. This drawback was reflected in the RMSE and ASE results provided by ArcGIS. However, the values of the MEs showed that the mean of the residuals was close to 0, except in the case of the β_counts_. The α_counts_ resulted in RMSE and ASE values of 7.2 and 9.4, respectively, which is noteworthy because the values for this measurement were low compared to the other parameters, indicating poorer measurement statistics. The values of the β/γ counts reflected a higher error, which could be explained by the influence of the background radiation around the probe used for the measurements. Comparison of the ArcGIS and NN estimates showed a similarity in their predictions. The NN predictions showed a positive bias in all six parameters as the ME values were positive. The CK predictions were dispersed as the ME values were both positive and negative. However, the root mean square error (RMSE) was lower for the NN predictions. The variability of the models was correctly assessed by both CK and NN as MSE ≈ 0 and RMSSE ≈ 1 were obtained. Therefore, the cross-validation of the two models was considered equivalent with no significant differences observed.

The graphic representation in ArcGIS is more versatile than that of the NN. However, as can be seen in Fig. 6, the architecture of the NN designed in this study allows the evaluation of the data through the creation of 3 clusters. The most interesting cluster was designated cluster -1, which showed that 32.6% of the values had a high heterogeneity. This percentage of data, as shown in Fig. 6, represents areas close to those with higher radioactivity, especially in the tailings area of the mine. However, clusters 0 and 1 grouped the loading area and the rest of the mine surface into 58.9% and 8.5%, respectively. This information is not as graphic in the case of ArcGIS.

The errors in the activity concentration prediction models for the 3 natural radioactive series were systematically lower for the NN than for the MRA, as observed in the RMSE bar diagrams in Fig. 7. The statistically significant parameters after the MRA were H*(10)_c+s_ and α_counts_ for the uranium and actinium series, and H*(10)_1 m_ and α_counts_ for the actinium series. The results for the uranium and actinium series again indicate the possible influence of background radiation, as the values of H*(10)_c+s_ and α_counts_ are less affected by background radiation due to their more focused measurements, the short range of alpha particles and the shielding used when measuring H*(10)_c+s_. The thorium series would be more affected by H*(10)_1 m_ and α_counts_, which may not reflect the reality of the study area, as the uranium and actinium series are two orders of magnitude higher than thorium. However, the Garson algorithm showed more variability in the factors influencing each of the radionuclides of the three natural radioactive series. Nevertheless, the Garson algorithm again agreed that α_counts_ are one of the factors most influencing the estimation of activity concentrations for all radionuclides except ^226^Ra. This discrepancy may be due to the fact that ^226^Ra was determined by suppressing the interference from ^235^U in the 186 keV photopeak, so it is not considered a single radionuclide in gamma spectrometry, unlike the other cases (Suárez-Navarro et al., 2018).

The validation showed that the radiological parameters (H*(10) and surface activity) were better estimated than the activity concentrations, as the relative biases were lower. The radiological parameters showed heteroscedastic variance, but the means were comparable in both cases (two-tailed Student's t-test for heteroscedastic variances). The NN achieved lower percentage differences than CK (70.8%). These results were not expected a priori, as CK obtained lower ME values when comparing the model estimates (Fig. 5). The results with higher relative bias were the α_counts_ and β_counts_ for validation point 1 (115 and 26,273 total counts per minute). This was due to the fact that the highest values for both parameters in the NN training and using the CK method were 82 and 11,210 total counts per minute, respectively, so these values were estimated outside the range of the experimental values. On the other hand, the means and variances of the activity concentrations determined from the radiological parameters estimated from the geographic coordinates were statistically equivalent to those obtained through gamma spectrometry. The best predictions were obtained for the actinium and thorium series, since the activity concentrations of their radionuclides were two orders of magnitude lower. The greater uncertainty observed in the activity concentrations compared to the radiological measurements can be explained by the inherent uncertainty of soil sampling (Glavič-Cindro et al., 2023).

## Conclusions

The estimates for the radiological parameters (H*(10)_c_, H*(10)_c+s_, H*(10)_1 m_, α_counts_, β_counts_ and β/γ_counts_) obtained using the neural network (NN) designed in this study were comparable to those obtained by CK. This result confirmed that the design of the NN using Convolutional Autoencoder (CAE) and Density-Based Spatial Clustering of Applications with Noise (DBSCAN) algorithms increased the accuracy of the predictions made by the NN. The NN is therefore an alternative to this software when it comes to characterising radiologically affected areas. The advantages of ArcGIS are too numerous to list, but the main advantage of NN is its ability to determine the percentages of values grouped by the DBSCAN algorithm. This makes it possible to assess potential uncertainties in the NN predictions, which are more difficult to interpret using CK. Increasing the number of experimental points would improve the accuracy of the predictions. However, the 141 *in-situ* measurements taken in this study are considered satisfactory from a real sampling perspective.

The validation of radiological values obtained using ArcGIS (CK) and NN compared with values obtained in situ was satisfactory for both models. However, the results obtained in the activity concentration estimation were less accurate than the gamma spectrometry results. Therefore, although it was confirmed that the laboratory measurements and the *in-situ* measurements were in agreement, the mathematical model estimation of activity concentrations falls short of the laboratory reality. It is clear that laboratory measurements will always be more accurate and precise than those estimated by mathematical prediction models. However, the results obtained in this study have shown that the models generated by CK, along with MRA or a NN, can yield results with satisfactory reproducibility. This allows for decisions that optimize the work of the laboratory and enhance the final conclusions of the research.

## Supplementary Information

Below is the link to the electronic supplementary material.

Supplementary file 1 (DOCX 244 kb)
