# Supplementary material for: Radiological characterization of the tailings of an abandoned copper mine using a neural network and geostatistical analysis through the Co-Kriging method
Source: Environ Geochem Health. 2024 Jul 9;46(8):297. doi: 10.1007/s10653-024-02070-8 (PMC11584432; doi:10.1007/s10653-024-02070-8)
Supplement: Supplementary file 1 — (DOCX 244 kb) [file 10653_2024_2070_MOESM1_ESM.docx]

**Table S.1.** Sampling points coordinates and values of dose rate measurements and α, β and β/γ activity counts measured in-situ

|  |  |  | Dose rate (nSv h^-1^) | | | Counts | | |
| --- | --- | --- | --- | --- | --- | --- | --- | --- |
| Code | Coordinates | | Contact + shielding | Contact | 1 m | α | β | β/γ |
| PT_0225_1 | 40°31'44.67"N | 3°59'38.38"O | 1100 | 1700 | 1200 | 74 | 11210 | 14810 |
| PT_0225_2 | 40°31'41.83"N | 3°59'38.33"O | 520 | 850 | 680 | 19 | 5419 | 8833 |
| PT_0302_1 | 40°31'41.84"N | 3°59'39.95"O | 80 | 270 | 320 | 9 | 1438 | 2597 |
| PT_0302_3 | 40°31'41.94"N | 3°59'40.35"O | 320 | 390 | 230 | 45 | 2253 | 3680 |
| PT_0302_4 | 40°31'42.05"N | 3°59'40.85"O | 240 | 470 | 210 | 8 | 2247 | 3990 |
| PT_0302_5 | 40°31'42.19"N | 3°59'41.07"O | 260 | 350 | 320 | 8 | 1852 | 3282 |
| PT_0302_6 | 40°31'42.81"N | 3°59'41.26"O | 260 | 390 | 400 | 4 | 1600 | 3248 |
| PT_0302_7 | 40°31'42.52"N | 3°59'40.88"O | 370 | 590 | 570 | 14 | 2852 | 5391 |
| PT_0302_8 | 40°31'42.33"N | 3°59'40.64"O | 350 | 380 | 350 | 26 | 2298 | 3368 |
| PT_0302_9 | 40°31'42.23"N | 3°59'39.94"O | 150 | 490 | 400 | 14 | 5593 | 3310 |
| PT_0304_1 | 40°31'44.36"N | 3°59'39.36"O | 200 | 320 | 290 | 5 | 1668 | 2699 |
| PT_0304_2 | 40°31'43.66"N | 3°59'39.48"O | 200 | 350 | 220 | 11 | 1850 | 2827 |
| PT_0304_3 | 40°31'43.92"N | 3°59'39.82"O | 210 | 290 | 190 | 7 | 1904 | 2571 |
| PT_0304_4 | 40°31'44.52"N | 3°59'40.11"O | 240 | 320 | 240 | 18 | 1605 | 2395 |
| PT_0304_5 | 40°31'44.04"N | 3°59'40.50"O | 240 | 270 | 230 | 4 | 1592 | 2597 |
| PT_0304_6 | 40°31'43.99"N | 3°59'40.82"O | 220 | 230 | 320 | 8 | 1484 | 2629 |
| PT_0304_7 | 40°31'43.97"N | 3°59'41.33"O | 380 | 790 | 390 | 34 | 4222 | 7652 |
| PT_0304_8 | 40°31'43.81"N | 3°59'41.00"O | 240 | 480 | 290 | 12 | 2300 | 3767 |
| PT_0309_1 | 40°31'44.02"N | 3°59'37.90"O | 610 | 900 | 430 | 11 | 3961 | 7839 |
| PT_0309_2 | 40°31'43.79"N | 3°59'37.76"O | 280 | 410 | 230 | 8 | 1550 | 3234 |
| PT_0309_3 | 40°31'43.57"N | 3°59'37.74"O | 280 | 320 | 320 | 9 | 1497 | 3005 |
| PT_0309_4 | 40°31'43.26"N | 3°59'37.51"O | 270 | 200 | 220 | 5 | 1550 | 2680 |
| PT_0309_5 | 40°31'42.98"N | 3°59'37.40"O | 220 | 370 | 270 | 6 | 1521 | 2845 |
| PT_0309_6 | 40°31'42.88"N | 3°59'37.86"O | 300 | 580 | 340 | 27 | 3326 | 5905 |
| PT_0309_7 | 40°31'42.64"N | 3°59'37.85"O | 230 | 250 | 290 | 10 | 1214 | 2438 |
| PT_0309_8 | 40°31'42.28"N | 3°59'37.85"O | 190 | 640 | 210 | 11 | 1667 | 3365 |
| PT_0309_9 | 40°31'42.00"N | 3°59'38.08"O | 260 | 350 | 210 | 3 | 1747 | 3643 |
| PT_0309_10 | 40°31'41.63"N | 3°59'38.27"O | 390 | 450 | 430 | 8 | 1855 | 3858 |
| PT_0309_11 | 40°31'41.81"N | 3°59'38.74"O | 220 | 350 | 250 | 5 | 1746 | 3213 |
| PT_0309_12 | 40°31'43.94"N | 3°59'38.23"O | 660 | 1500 | 100 | 68 | 6364 | 11110 |
| PT_0309_13 | 40°31'43.74"N | 3°59'38.01"O | 370 | 680 | 350 | 12 | 2241 | 4530 |
| PT_0309_14 | 40°31'43.87"N | 3°59'38.55"O | 250 | 600 | 420 | 10 | 2937 | 5592 |
| PT_0309_15 | 40°31'43.64"N | 3°59'38.30"O | 280 | 410 | 260 | 2 | 1295 | 2775 |
| PT_0311_1 | 40°31'43.21"N | 3°59'40.91"O | 320 | 540 | 350 | 21 | 2992 | 5116 |
| PT_0311_2 | 40°31'43.46"N | 3°59'40.94"O | 250 | 400 | 420 | 5 | 2205 | 4080 |
| PT_0311_3 | 40°31'43.65"N | 3°59'41.09"O | 490 | 1000 | 730 | 15 | 3835 | 6911 |
| PT_0311_4 | 40°31'43.40"N | 3°59'41.30"O | 350 | 510 | 470 | 10 | 2475 | 4365 |
| PT_0311_5 | 40°31'43.29"N | 3°59'41.14"O | 410 | 920 | 870 | 14 | 3775 | 6998 |
| PT_0311_6 | 40°31'43.10"N | 3°59'41.23"O | 510 | 1100 | 710 | 15 | 4364 | 8283 |
| PT_0311_7 | 40°31'42.94"N | 3°59'41.18"O | 570 | 810 | 540 | 21 | 4582 | 8010 |
| PT_0311_8 | 40°31'42.88"N | 3°59'40.91"O | 320 | 660 | 750 | 7 | 2781 | 5461 |
| PT_0311_9 | 40°31'42.93"N | 3°59'40.60"O | 270 | 470 | 510 | 8 | 3305 | 5525 |
| PT_0311_10 | 40°31'43.04"N | 3°59'40.31"O | 350 | 680 | 580 | 12 | 3436 | 6166 |
| PT_0311_11 | 40°31'43.02"N | 3°59'40.09"O | 270 | 840 | 570 | 13 | 4138 | 6827 |
| PT_0311_12 | 40°31'43.08"N | 3°59'39.68"O | 620 | 870 | 790 | 18 | 4986 | 8836 |
| PT_0311_13 | 40°31'42.77"N | 3°59'40.51"O | 220 | 390 | 290 | 8 | 1778 | 3610 |
| PT_0311_14 | 40°31'42.87"N | 3°59'40.16"O | 260 | 300 | 270 | 7 | 1395 | 3039 |
| PT_0311_15 | 40°31'42.87"N | 3°59'39.71"O | 190 | 490 | 420 | 12 | 1865 | 3805 |
| PT_0311_16 | 40°31'42.93"N | 3°59'39.17"O | 180 | 370 | 370 | 6 | 1398 | 3072 |
| PT_0311_17 | 40°31'42.97"N | 3°59'38.20"O | 230 | 350 | 390 | 10 | 1774 | 3715 |
| PT_0311_18 | 40°31'42.96"N | 3°59'38.76"O | 190 | 340 | 320 | 8 | 1140 | 2640 |
| PT_0311_19 | 40°31'42.73"N | 3°59'38.50"O | 260 | 440 | 250 | 8 | 1474 | 2963 |
| PT_0311_20 | 40°31'42.60"N | 3°59'39.14"O | 260 | 240 | 350 | 16 | 1404 | 2854 |
| PT_0311_21 | 40°31'42.10"N | 3°59'39.32"O | 180 | 410 | 300 | 9 | 1515 | 3422 |
| PT_0311_22 | 40°31'41.91"N | 3°59'38.91"O | 290 | 400 | 290 | 6 | 1580 | 3493 |
| PT_0311_23 | 40°31'41.82"N | 3°59'38.54"O | 260 | 370 | 260 | 7 | 1716 | 3228 |
| PT_0311_24 | 40°31'42.18"N | 3°59'38.17"O | 220 | 430 | 240 | 11 | 1933 | 3992 |
| PT_0311_25 | 40°31'42.75"N | 3°59'40.89"O | 380 | 730 | 580 | 12 | 3371 | 6504 |
| PT_0315_1 | 40°31'43.26"N | 3°59'39.88"O | 430 | 890 | 730 | 15 | 3815 | 7533 |
| PT_0315_2 | 40°31'43.16"N | 3°59'40.03"O | 510 | 950 | 710 | 8 | 3475 | 7620 |
| PT_0315_3 | 40°31'43.05"N | 3°59'40.34"O | 510 | 770 | 660 | 19 | 4982 | 8196 |
| PT_0315_4 | 40°31'43.02"N | 3°59'40.55"O | 390 | 680 | 510 | 18 | 2983 | 6064 |
| PT_0315_5 | 40°31'43.23"N | 3°59'40.38"O | 540 | 890 | 610 | 22 | 4493 | 7941 |
| PT_0315_6 | 40°31'43.31"N | 3°59'40.00"O | 640 | 950 | 750 | 8 | 4465 | 8237 |
| PT_0315_7 | 40°31'43.27"N | 3°59'40.10"O | 570 | 810 | 750 | 15 | 4490 | 8061 |
| PT_0315_8 | 40°31'43.44"N | 3°59'40.07"O | 210 | 300 | 380 | 8 | 1131 | 3126 |
| PT_0315_9 | 40°31'43.57"N | 3°59'40.24"O | 450 | 1200 | 700 | 24 | 4333 | 8537 |
| PT_0315_10 | 40°31'43.49"N | 3°59'40.41"O | 260 | 260 | 350 | 2 | 1176 | 2979 |
| PT_0315_11 | 40°31'43.18"N | 3°59'40.66"O | 270 | 490 | 410 | 10 | 1940 | 4126 |
| PT_0315_12 | 40°31'42.97"N | 3°59'40.84"O | 260 | 640 | 430 | 22 | 3540 | 5658 |
| PT_0315_13 | 40°31'43.31"N | 3°59'40.88"O | 250 | 650 | 470 | 24 | 3716 | 5934 |
| PT_0315_14 | 40°31'43.45"N | 3°59'40.93"O | 250 | 370 | 410 | 10 | 1773 | 3951 |
| PT_0315_15 | 40°31'43.74"N | 3°59'40.95"O | 200 | 440 | 390 | 8 | 1947 | 3900 |
| PT_0315_16 | 40°31'43.90"N | 3°59'41.06"O | 180 | 340 | 300 | 9 | 1412 | 3141 |
| PT_0315_17 | 40°31'43.49"N | 3°59'39.63"O | 260 | 370 | 460 | 9 | 1910 | 3734 |
| PT_0315_18 | 40°31'43.33"N | 3°59'39.47"O | 270 | 380 | 320 | 5 | 1620 | 2970 |
| PT_0315_19 | 40°31'43.74"N | 3°59'39.35"O | 180 | 370 | 270 | 5 | 1374 | 2796 |
| PT_0315_20 | 40°31'43.49"N | 3°59'39.05"O | 260 | 340 | 150 | 11 | 1400 | 2860 |
| PT_0315_21 | 40°31'43.72"N | 3°59'38.78"O | 260 | 350 | 270 | 4 | 1431 | 3151 |
| PT_0315_22 | 40°31'43.53"N | 3°59'38.56"O | 320 | 210 | 340 | 4 | 1290 | 2584 |
| PT_0315_23 | 40°31'42.90"N | 3°59'37.90"O | 210 | 260 | 280 | 14 | 1227 | 2580 |
| PT_0315_24 | 40°31'42.46"N | 3°59'37.88"O | 210 | 280 | 210 | 10 | 1247 | 2435 |
| PT_0323_1 | 40°31'43.94"N | 3°59'37.55"O | 470 | 1200 | 660 | 30 | 5245 | 10585 |
| PT_0323_2 | 40°31'43.82"N | 3°59'37.46"O | 1500 | 2000 | 920 | 82 | 10060 | 16471 |
| PT_0323_3 | 40°31'43.79"N | 3°59'37.28"O | 870 | 1500 | 820 | 62 | 7656 | 13727 |
| PT_0323_4 | 40°31'43.72"N | 3°59'37.00"O | 800 | 1100 | 730 | 23 | 4049 | 8698 |
| PT_0323_5 | 40°31'43.61"N | 3°59'36.84"O | 450 | 580 | 350 | 12 | 2474 | 5309 |
| PT_0323_6 | 40°31'43.59"N | 3°59'36.67"O | 600 | 1200 | 520 | 17 | 4339 | 8447 |
| PT_0323_7 | 40°31'43.54"N | 3°59'36.37"O | 430 | 860 | 320 | 11 | 2697 | 5905 |
| PT_0323_8 | 40°31'43.25"N | 3°59'36.44"O | 190 | 300 | 370 | 5 | 1345 | 2845 |
| PT_0323_9 | 40°31'42.75"N | 3°59'36.36"O | 250 | 280 | 260 | 6 | 909 | 2289 |
| PT_0323_10 | 40°31'43.41"N | 3°59'36.04"O | 190 | 260 | 240 | 8 | 1222 | 2867 |
| PT_0323_11 | 40°31'44.11"N | 3°59'36.39"O | 240 | 320 | 290 | 12 | 1438 | 3038 |
| PT_0323_12 | 40°31'44.12"N | 3°59'36.87"O | 240 | 320 | 240 | 15 | 1405 | 3241 |
| PT_0323_13 | 40°31'44.53"N | 3°59'37.54"O | 190 | 230 | 270 | 6 | 1190 | 2704 |
| PT_0323_14 | 40°31'44.40"N | 3°59'37.66"O | 190 | 430 | 320 | 9 | 2124 | 4360 |
| PT_0323_15 | 40°31'44.41"N | 3°59'37.92"O | 260 | 540 | 430 | 15 | 2604 | 4552 |
| PT_0323_16 | 40°31'44.58"N | 3°59'38.07"O | 770 | 1500 | 1100 | 43 | 7371 | 13250 |
| PT_0323_17 | 40°31'44.69"N | 3°59'38.30"O | 280 | 350 | 540 | 14 | 2086 | 4039 |
| PT_0323_18 | 40°31'44.84"N | 3°59'38.30"O | 160 | 260 | 380 | 6 | 1596 | 3235 |
| PT_0323_19 | 40°31'44.89"N | 3°59'38.62"O | 210 | 260 | 260 | 10 | 1267 | 2515 |
| PT_0323_20 | 40°31'45.04"N | 3°59'38.86"O | 190 | 250 | 270 | 1 | 1296 | 2877 |
| PT_0323_21 | 40°31'44.64"N | 3°59'38.57"O | 230 | 410 | 340 | 5 | 1769 | 3747 |
| PT_0323_22 | 40°31'44.27"N | 3°59'38.29"O | 160 | 250 | 320 | 10 | 1363 | 3076 |
| PT_0323_23 | 40°31'44.30"N | 3°59'38.03"O | 150 | 340 | 320 | 4 | 1275 | 2836 |
| PT_0323_24 | 40°31'43.93"N | 3°59'38.22"O | 260 | 660 | 820 | 25 | 3883 | 6555 |
| PT_0323_25 | 40°31'43.57"N | 3°59'37.75"O | 160 | 320 | 410 | 4 | 1465 | 3052 |
| PT_0323_26 | 40°31'43.17"N | 3°59'37.51"O | 230 | 280 | 180 | 1 | 1235 | 2480 |
| PT_0325_1 | 40°31'42.72"N | 3°59'41.48"O | 280 | 410 | 350 | 5 | 1695 | 3682 |
| PT_0325_2 | 40°31'42.50"N | 3°59'41.57"O | 210 | 280 | 350 | 7 | 1575 | 3315 |
| PT_0325_3 | 40°31'42.54"N | 3°59'42.19"O | 200 | 300 | 280 | 15 | 1445 | 3047 |
| PT_0325_4 | 40°31'42.31"N | 3°59'41.30"O | 320 | 270 | 240 | 10 | 1943 | 3705 |
| PT_0325_5 | 40°31'42.21"N | 3°59'41.57"O | 220 | 470 | 250 | 8 | 2101 | 4118 |
| PT_0325_6 | 40°31'41.89"N | 3°59'41.32"O | 160 | 320 | 350 | 3 | 1641 | 3261 |
| PT_0325_7 | 40°31'41.66"N | 3°59'41.11"O | 160 | 200 | 260 | 5 | 1415 | 2986 |
| PT_0325_8 | 40°31'41.54"N | 3°59'40.64"O | 220 | 270 | 300 | 8 | 1634 | 3532 |
| PT_0325_9 | 40°31'41.85"N | 3°59'40.88"O | 320 | 380 | 400 | 5 | 1865 | 3841 |
| PT_0325_10 | 40°31'41.15"N | 3°59'40.74"O | 260 | 210 | 250 | 4 | 1248 | 2749 |
| PT_0325_11 | 40°31'41.75"N | 3°59'40.15"O | 200 | 420 | 270 | 9 | 1744 | 3596 |
| PT_0325_12 | 40°31'41.21"N | 3°59'39.71"O | 210 | 350 | 340 | 6 | 1361 | 2527 |
| PT_0325_13 | 40°31'41.74"N | 3°59'39.48"O | 240 | 390 | 240 | 6 | 1486 | 3267 |
| PT_0325_14 | 40°31'41.55"N | 3°59'38.81"O | 240 | 320 | 350 | 3 | 1712 | 3199 |
| PT_0325_15 | 40°31'41.51"N | 3°59'38.32"O | 350 | 280 | 210 | 7 | 1910 | 3799 |
| PT_0325_16 | 40°31'42.04"N | 3°59'37.40"O | 280 | 240 | 200 | 3 | 1222 | 2519 |
| PT_0325_17 | 40°31'42.77"N | 3°59'37.37"O | 160 | 260 | 240 | 8 | 1347 | 2699 |
| PT_0325_18 | 40°31'42.34"N | 3°59'37.08"O | 230 | 260 | 240 | 8 | 1366 | 2769 |
| PT_0325_19 | 40°31'41.97"N | 3°59'36.84"O | 160 | 290 | 370 | 8 | 1209 | 2591 |
| PT_0325_20 | 40°31'41.59"N | 3°59'36.62"O | 280 | 240 | 190 | 5 | 1272 | 2591 |
| PT_0325_21 | 40°31'42.56"N | 3°59'40.03"O | 440 | 370 | 260 | 6 | 1613 | 3484 |
| PT_0506_1 | 40°31'42.47"N | 3°59'40.51"W | 250 | 160 | 350 | 6 | 1358 | 2832 |
| PT_0506_2 | 40°31'43.16"N | 3°59'41.45"W | 450 | 820 | 830 | 21 | 4159 | 7599 |
| PT_0506_3 | 40°31'43.30"N | 3°59'41.64"W | 240 | 300 | 320 | 4 | 1533 | 3054 |
| PT_0506_4 | 40°31'43.49"N | 3°59'41.58"W | 160 | 380 | 290 | 3 | 1504 | 2997 |
| PT_0506_5 | 40°31'42.26"N | 3°59'39.19"W | 160 | 250 | 240 | 4 | 1412 | 2762 |
| PT_0506_6 | 40°31'42.41"N | 3°59'39.07"W | 220 | 320 | 230 | 1 | 1367 | 2871 |
| PT_0506_7 | 40°31'42.37"N | 3°59'38.57"W | 290 | 470 | 290 | 7 | 1474 | 2969 |
| PT_0506_8 | 40°31'42.56"N | 3°59'38.75"W | 220 | 160 | 290 | 6 | 1336 | 2899 |
| PT_0506_9 | 40°31'42.32"N | 3°59'38.88"W | 250 | 320 | 370 | 18 | 1451 | 2804 |
| PT_0506_10 | 40°31'42.81"N | 3°59'38.72"W | 290 | 300 | 280 | 7 | 1532 | 2857 |
| PT_0506_11 | 40°31'42.41"N | 3°59'39.42"W | 200 | 410 | 240 | 2 | 1479 | 2270 |
| PT_0506_12 | 40°31'42.58"N | 3°59'39.49"W | 160 | 320 | 240 | 5 | 1662 | 3276 |

**Table S.2.** Sampling points coordinates and gross α activity and gross β activity determined in the laboratory

| Code | Coordinates | | Gross α Activity  (Bq kg^-1^) | | Gross β Activity  (Bq kg^-1^) |
| --- | --- | --- | --- | --- | --- |
| PT_0225_1 | 40°31’44.67”N | 3°59’38.38”O | | 22874 ± 2629 | 7634 ± 620 |
| PT_0225_2 | 40°31’41.83”N | 3°59’38.33”O | | 13376 ± 1548 | 5206 ± 399 |
| PT_0302_7 | 40°31’42.52”N | 3°59’40.88”O | | 5495 ± 648 | 2549 ± 192 |
| PT_0302_8 | 40°31’42.33”N | 3°59’40.64”O | | 2269 ± 281 | 2219 ± 143 |
| PT_0311_3 | 40°31’43.65”N | 3°59’41.09”O | | 9257 ± 1079 | 6431 ± 401 |
| PT_0311_25 | 40°31’42.75”N | 3°59’40.89”O | | 7890 ± 921 | 5760 ± 355 |
| PT_0315_2 | 40°31’43.16”N | 3°59’40.03”O | | 4030 ± 482 | 3545 ± 221 |
| PT_0315_9 | 40°31’43.57”N | 3°59’40.24”O | | 12321 ± 1426 | 8130 ± 512 |
| PT_0315_13 | 40°31’43.31”N | 3°59’40.88”O | | 9849 ± 1144 | 6750 ± 425 |
| PT_0323_2 | 40°31’43.82”N | 3°59’37.46”O | | 58095 ± 6646 | 28432 ± 1901 |
| PT_0323_5 | 40°31’43.61”N | 3°59’36.84”O | | 4205 ± 501 | 3137 ± 201 |
| PT_0323_16 | 40°31’44.58”N | 3°59’38.07”O | | 20565 ± 2369 | 12391 ± 786 |
| PT_0323_24 | 40°31’43.93”N | 3°59’38.22”O | | 12221 ± 1415 | 7514 ± 484 |
| PT_0325_8 | 40°31’41.54”N | 3°59’40.64”O | | 1535 ± 200 | 2102 ± 132 |
| PT_0325_21 | 40°31’42.56”N | 3°59’40.03”O | | 1080 ± 149 | 1856 ± 116 |
| PT_0323_17 | 40°31’44.69”N | 3°59’38.30”O | | 4436 ± 541 | 2650 ± 178 |
| PT_0323_14 | 40°31’44.40”N | 3°59’37.66”O | | 2852 ± 348 | 2698 ± 181 |
| PT_0309_15 | 40°31’43.64”N | 3°59’38.30”O | | 634 ± 77 | 1645 ± 110 |
| PT_0315_7 | 40°31’43.27”N | 3°59’40.10”O | | 4753 ± 579 | 5703 ± 383 |
| PT_0315_3 | 40°31’43.05”N | 3°59’40.34”O | | 6020 ± 734 | 6328 ± 425 |
| PT_0311_25 | 40°31’42.75”N | 3°59’40.89”O | | 3802 ± 463 | 4282 ± 287 |
| PT_0311_15 | 40°31’42.87”N | 3°59’39.71”O | | 3802 ± 463 | 2369 ± 159 |
| PT_0302_7 | 40°31’42.52”N | 3°59’40.88”O | | 4436 ± 541 | 3622 ± 243 |
| PT_0506_8 | 40°31’42.56”N | 3°59’38.75”W | | 1901 ± 232 | 1697 ± 114 |

**Table S.3.** Gamma spectrometry determined in the laboratory

**Figure S.1**. Validation of radiological parameters (H*(10)_c_, H*(10)_c+s_, H*(10)_1m_, α_counts_, β_counts_ and β/γ_counts_) and activity concentrations estimated from ArcGIS, MRA and the NNs in 4 verification samples.
